# The Path to Global Equity in Surgical Care: Clinical and Technical Results of Cross-Border, Ultra-Long-Distance, Asia to Middle East Human Telesurgery

**DOI:** 10.1590/S1677-5538.IBJU.2025.0331

**Published:** 2025-07-30

**Authors:** Saad Aldousari, Ahmad Almarzouq, Abdelrahman Eltafahny, Saud Alhelal, Wang Jiayin, Abdelkareem Hassan, Ahmed Shahin, Saleh Bubishate, Basmah Bahbahani

**Affiliations:** 1 Sabah Al-Ahmad Urology Center Department of Surgery (Urology) Kuwait Department of Surgery (Urology), Sabah Al-Ahmad Urology Center, Kuwait;; 2 Kuwait University Department of Surgery (Urology Division) Faculty of Medicine Kuwait Department of Surgery (Urology Division), Faculty of Medicine, Kuwait University, Kuwait;; 3 The University of Texas MD Anderson Cancer Center Department of Urology Houston TX USA Department of Urology, The University of Texas MD Anderson Cancer Center, Houston, TX, USA;; 4 MicroPort MedBot Ltd Department of Telecommunications and Algorithm Control Shanghai China Department of Telecommunications and Algorithm Control, MicroPort MedBot Ltd, Shanghai, China;; 5 Kuwait Institute for Medical Specialization Urology Faculty Kuwait City Kuwait Urology Faculty, Kuwait Institute for Medical Specialization, Kuwait City, Kuwait

**Keywords:** Robotic Surgical Procedures, Minimally Invasive Surgical Procedures, Prostatectomy

## Abstract

**Purpose:**

To demonstrate the feasibility and reproducibility of Ultra-long-distance, Asia to Middle East human telesurgery using low latency connectivity on multiple patients and different procedures.

**Methods:**

Five human telesurgeries were performed over six months using a multiport robotic platform with fiber-optic and 5G connection. Remote surgeon (SA) was in Shanghai, China, and patients were in Kuwait City, Kuwait 7,000 kilometers (Km) apart.

**Results:**

Case 1 was performed in December 2024. Cases 2 – 5 were performed consecutively over four days in April 2025. Three robotic assisted radical prostatectomies and two robotic assisted partial nephrectomies were performed. There were no clinical or technical issues encountered during any of the procedures. The average round-trip latency for all procedures was 166.6 milliseconds (ms) using fiber-optic broadband network with 5G network as back-up. Enhanced cybersecurity was employed with no recorded threats. There was an experienced robotic surgeon (AA) present in the operating room in Kuwait with a clear criteria for console take-over in case of emergencies. There were no reported complications. Patients were discharged on post-operative day two and three. Final pathology showed low grade, low stage disease in all patients and no evidence of disease at eight weeks follow-up.

**Conclusion:**

This study demonstrated the feasibility and reproducibility of multiple human telesurgical procedures through successful collaboration between two countries using a robotic system with telecommunication capabilities, with good outcomes and without complications. However, there is a need to establish robust international guidelines to allow globalization of telesurgery.

## INTRODUCTION

Telesurgery, an innovation that allows surgeons to perform procedures remotely using advanced robotic technology, has revolutionized the way surgical care is delivered (1). From a concept born in the military, telesurgery has progressed over the past two decades with the aim to provide expert surgical care to distant and underserved locations by overcoming geographical barriers, improving global education and mentorship, and increasing surgical accuracy and safety (2). Its application could also help in delivering surgical care in extreme clinical setting such as battlefields, natural disasters, humanitarian assistance missions, underwater missions, and even space missions (3).

Since the groundbreaking Lindbergh Operation in 2001 where Marescaux et al. and his group demonstrated the feasibility of transatlantic human cholecystectomy from New York City (USA) to Strasbourg (France) spanning a distance of 6200 kilometers (Km), the widespread adoption of remote surgery remained limited due to issues relating to network reliability for long distances, lack of training, poor robotic precision, high cost, and cybersecurity concerns (4, 5). However, recent developments in high speed 5G and fiber-optic networks, surgeons experience, audiovisual communications, secure data transmission, and cost-effective robotic platforms with built-in remote connectivity capabilities allowed us to overcome many of the challenges that previously limited telesurgery (6). Prior to globalization of telesurgery, there exists a need for standardized guidelines to address the grey areas surrounding it including critical ethical concerns, liability issues, infrastructure requirements, and training and certification protocols (7).

In recent years, several published trials have described telesurgery in human and animal models between continents and different time zones (1, 6, 8-12). Those were considered promising progressive steps forward towards universal healthcare access, providing a template for the telesurgical community to benefit from. In December 2024, our group demonstrated the feasibility of performing ultra-long distance Asia to Middle East human robotic assisted radical prostatectomy (RARP) marking the first telesurgery in the Middle East (12). The surgeon (SA) was in Shanghai, China, using an immersive surgeon console to control a multiport robotic platform docked on a patient 7,000 Km away in Kuwait City.

Telesurgery provides the context for global medical collaboration and telementoring (13, 14). Through advanced communication technologies, surgeons from various geographical locations can consult or jointly conduct surgeries in real-time. This has been shown using animal models, spanning the longest recorded distance of 13,000 Km (Orland, USA to Shanghai, China) with a mean latency of 139 milliseconds (ms) and the ability to switch console control multiple times during surgeries. Surgeons could communicate and discuss cases in real-time and transfer control when needed (15)

Robotic assisted surgery is expanding in the Middle East and the Gulf Cooperation Council (GCC), fueled by investments in technology and growing expertise. Telesurgery fits perfectly with this expansion to address disparities in access to advanced surgical care by connecting surgeons in remote or poorly equipped areas with experts across the region and enhance skill and confidence by providing tele-mentoring. The current study had two primary endpoints. First, pilot demonstration of the feasibility and reproducibility of multiple ultra-long-distance human telesurgeries over different urological procedures with optimal network latency and acceptable outcomes. Secondly, to demonstrate the feasibility of transcontinental tele-proctoring in human model. To our knowledge this is the largest report of telesurgery cases with short-term outcomes in the Middle East, with demonstration of tele-mentoring in human model.

## MATERIALS AND METHODS

### Patient and hospital details

A telesurgery review committee was established at our center to ensure readiness and optimization of our project (12). Members of this committee include: hospital director, head of department of surgery, anesthesiology, nursing, and biomedical engineering including remote and local telecommunication specialists. Patients’ demographics and pre-operative clinical data are shown in Table-1. The robot used in the present study was the MicroPort Toumai robotic system® (MTRS). It was capable with fiber-optic, 5G, and wireless network connections. It had national medical products administration (NMPA) and Conformite European (CE) approvals (13, 16). The MTRS was very similar to the Da Vinci Xi® in terms of the console and patient cart design making an easy transition for the surgeons using it (17-20). RARP and RAPN were performed according to our previously described technique (12, 21). The hospital in Kuwait was a tertiary urology referral center with experience in robotic surgery since 2014 where the first robotic procedure was performed in the country, and more than 2000 local robotic urological procedures (mostly oncological) performed at the center to date. Both the remote and local surgeons were North American board certified, fellowship trained uro-oncologists. The remote surgeon (SA) was located at MTRS headquarters in Shanghai, China, and the local robotic surgical team lead by the local surgeon (AA) were located in the operating room in Kuwait. The team was capable of inserting trocars, docking the robot, and inserting/exchanging instruments. The contingency plan was set for the local team to take over and finish the case if any of the following were to happen: patient hemodynamic instability, latency > 300 ms lasting for > 5 minutes, network failure with loss of video/audio feeds, suspected or proven cyber-attack, intraoperative complications, and/or technical problems with the robotic surgical system. In case there was a need to take-over due to any of the aforementioned criteria, the local surgeon has the ability to immediately assume full control of the MTRS away from the remote surgeon using the local console. In the event of technical problems with the MTRS, the local surgeon could exchange to the local Da Vinci Xi® robotic system or convert to laparoscopy or open approach if they deemed necessary (12)

**Table 1:** Patient demographics and pre-operative data.

	Dec 20^th^, 2024, RARP/Case 1	April 10^th^, 2025, RAPN/Case 2	April 11^th^, 2025, RAPN/Case 3	April 12^th^, 2025, RARP/Case 4	April 13^th^, 2025, RARP/Case 5
**Age**	68	56	51	57	59
**Sex**	M	F	M	M	M
**BMI (kg/m**(2)	30.1	36.7	32.9	31.5	29.6
**PIRADS**	3	N/A	N/A	5	5
**PSA (ng/mL)**	6.8	N/A	N/A	7.2	7.69
**Prostate size (grams)**	55	N/A	N/A	37	41
**PSA density (ng/mL**(2)	0.08	N/A	N/A	0.18	0.18
**Clinical T stage**	cT1c	cT1a	cT1a	cT1c	cT1c
**ISUP-GG**	10 cores of 3+3 GG1	N/A	N/A	4 cores of 3+4 2 cores of 3+3 GG2	6 cores of 3+4 6 cores of 3+3 GG2
**Briganti score**	4.1%	N/A	N/A	4.9%	6%
**Decision to do lymph node dissection**	No	N/A	N/A	Yes	Yes
**SHIM score**	<7	N/A	N/A	<7	<7
**RENAL nephrometry score**	N/A	5a	6p	N/A	N/A

RAPN = robotic assisted partial nephrectomy; RARP = robotic assisted radical prostatectomy; BMI = body mass index; PIRADS = prostate imaging reporting and data system; N/A = not applicable; PSA = prostate specific antigen; ISUP-GG = international society of uro-pathology grade group; SHIM = sexual health inventory for men

### Informed consent and ethical approval

The research project received institutional review board (IRB) approval. A detailed informed consent was obtained from all patients in this study with clear discussion of risks, possible complications, and alternatives. All patients met with telesurgery review committee members and fully understood the role of the remote and local teams. In case of adverse events the responsibility falls on the Kuwaiti team including the remote surgeon (SA) who was the primary urologist for all the patients (10)

### Telesurgery and network details

On the Kuwaiti end, a primary dedicated fiber-optic broadband network was used with a redundancy 5G network as back-up. We added next generation firewall and high-end load balancer for added cybersecurity. On the Shanghai end, two dedicated fiber-optic broadband networks, a primary and a back-up, were employed to secure a redundancy network in case of primary network failure (22). Multiple connectivity testing was performed on a regular basis prior to commencing the project to ensure an acceptable Round-trip latency (RTL) between Shanghai and Kuwait. This averaged at 164 ms which was well within the acceptable range not to interfere with the smooth going of the procedure (23, 24)

**Figure 1:**
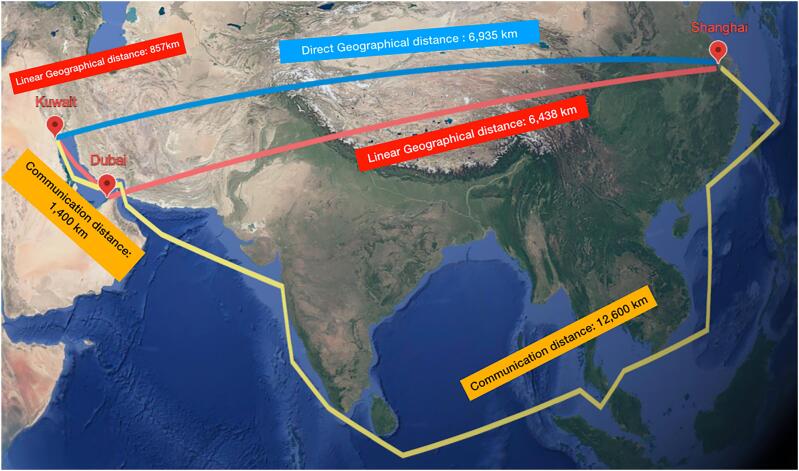
Round trip communication distance from Shanghai to Kuwait. Relay server was in Shanghai and connected to Dubai using international ethernet private line.

**Figure 2:**
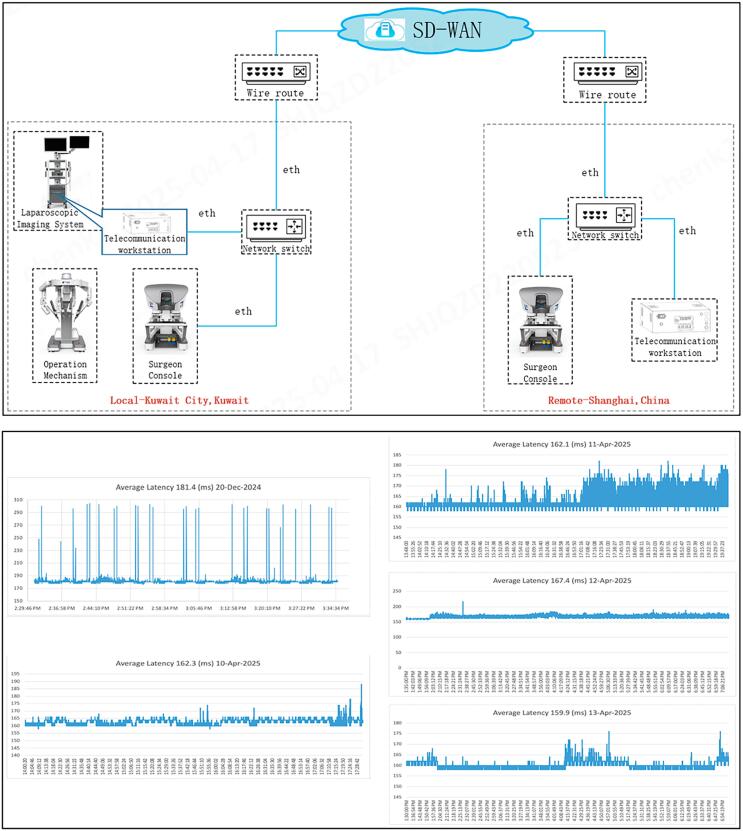
Schematic network setup between Kuwait and Shanghai showing ethernet in the hospital connected to software-defined wide-area-network cloud server in Shanghai. One-hour delay display in milliseconds for the five cases, with whole procedure average latency for each procedure.

The exact linear geographical distance between the hospital in Kuwait City and MTRS headquarters in Shanghai was 6,935 Km (Figure-1). A local relay server was setup in Shanghai, China, and both ends communicated with each other by connecting to this relay server. Based on the international ethernet private line (IEPL) dedicated fiber-optic between Dubai, United Arab Emirates and Shanghai, China, a software-defined wide-area-network (SD-WAN) technology (a virtual cloud architecture with its own firewall) was adopted to reduce network delay, add more security, and stabilize network communication (Figure-2(7). Communications between both sides of the connection were encrypted. A control request with the correct access code from the remote surgeon (SA) in Shanghai, China, was required with approval from the local surgeon (AA) in the operating room in Kuwait City. A separate line of audio-visual communication between the remote surgeon and the local team in Kuwait was created as well (1). Both teams had access to the patient's clinical information. Communication was in the English language. This set up was essential to allow evaluation of surgical safety, latency performance, and network stability which was the main objectives of the current study.

SD-WAN = software-defines wide-area-network; eth = ethernet. ms = milliseconds

## RESULTS

### Peri-operative data

Post-operative clinical and network data are shown in Table-2. There were two RAPN and three RARP. Case 1 was performed on a Friday, and cases 2 – 5 were performed on Thursday, Friday, Saturday, and Sunday which resulted in the avoidance of network congestion (Figure-2). Each procedure started at seven o'clock in the morning Kuwait standard time (equivalent to noon Shanghai standard time). There were no peri-operative complications recorded. None of the patients required a blood transfusion. All patients were discharged on the second post-operative day, except one of the RAPNs, where the patient was discharged on the third post-operative day for blood pressure monitoring as she was known to have hypertension with no interventions needed. The Foley catheter was removed for the RARP patients on the 10th post-operative day. Resection surgical margins were negative for all patients. Patients were seen at eight weeks post-operatively with no evidence of disease on imaging, a stable renal function (RAPN patients), an undetectable PSA (RARP patients), and no short-term complications.

**Table. 2:** Post-operative clinical and telecommunication data.

		Dec 20^th^, 2024, RARP/Case 1	April 10^th^, 2025, RAPN/Case 2	April 11^th^, 2025, RAPN/Case 3	April 12^th^, 2025, RARP/Case 4	April 13^th^, 2025, RARP/Case 5
**Surgeon to patient distance**		6,935 km	6,935 km	6,935 km	6,935 km	6,935 km
**Latency**						
	**Average (ms)**	181.1	162.3	162.1	167.4	159.9
	Highest (ms)	304	188	182	216	176
	Lowest (ms)	176	158	158	158	158
Console time (min)		210	180	250	180	300
Warm ischemia time (min)		N/A	25	22	N/A	N/A
**Hemoglobin (g/dL)**						
	Pre-operative	12.9	12.8	15.1	13.4	12.5
	Post-operative	12	11.1	15	12.5	11.9
**PSA ng/mL (8 Weeks post-operative)**		<0.01	N/A	N/A	<0.01	<0.01
**Creatinine** μ**mol/L (eGFRmL/min/1.73m²)**						
	Pre-operative	N/A	53 (102)	78 (98)	N/A	N/A
	Post-operative	N/A	58 (98)	80 (97)	N/A	N/A
**ICU admission**		No	No	No	No	No
**Estimated blood loss (mL)**		70	100	120	20	100
**Blood transfusion**		No	No	No	No	No
**Post-operative fever**		No	No	No	No	No
**Readmission**		No	No	No	No	No
**Complications (Clavien-Dindo grade)**		None	None	None	None	None
**Pathological stage (TNM)**		pT2 Nx Mx	pT1a Nx Mx	pT1a Nx Mx	pT3a N0 M0	pT3a N0 M0
**ISUP-GG**		GG 2	N/A	N/A	GG 2	GG 2
**Extracapsular extension**		Yes	N/A	N/A	Yes	Yes
**Fuhrman grade**		N/A	1	2	N/A	N/A
**Histology**		Adenocarcinoma	Clear cell renal cell carcinoma	Clear cell renal cell carcinoma	Adenocarcinoma	Adenocarcinoma
**Follow-up imaging**		N/A	U/S: No evidence of disease	U/S: No evidence of disease	Cystogram Day 10: No leak	Cystogram Day 10: No leak

RAPN = robotic assisted partial nephrectomy; RARP = robotic assisted radical prostatectomy; ms = milliseconds; min = minute; PSA = prostate specific antigen; GFR = glomerular filtration rate; ICU = intensive care unit; mL = millilitres; ISUP-GG = international society of uro-pathology grade group; N/A = not applicable; U/S = ultrasound abdomen and pelvis

**Figure 3:**
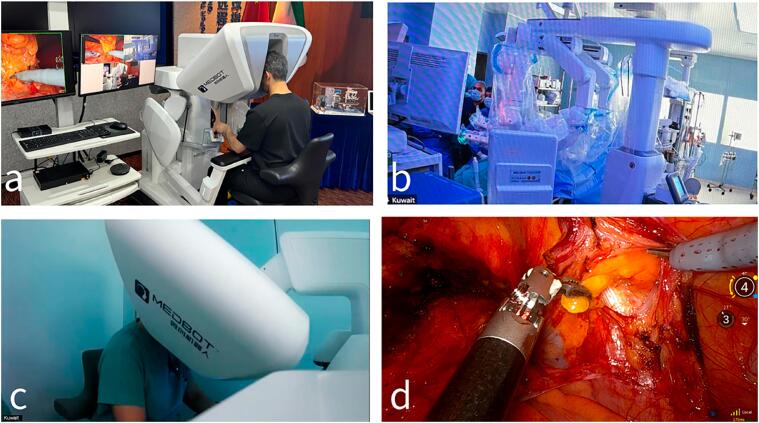
a) Remote surgeon using the MedBot Toumai console while performing hilar dissection during right sided RAPN. b) Patient-side cart docked during right sided RAPN. c) Robotic fellow using the local console (Kuwait) while being tele-mentored by the remote surgeon (Shanghai). d) right sided standard pelvic lymph node dissection during tele-mentoring showing a round-trip latency of 170 ms.

During cases 4 and 5 (RARP), we conducted a tele-mentoring experience where a local robotic fellow (SB) in Kuwait performed unilateral right standard pelvic lymph node dissection under supervision of the remote surgeon (SA) (Figure-3). The duration of the telementoring was 25 and 30 minutes, respectively. There was smooth switching of the console between the proctor and the fellow multiple times for directions only without take-over, with clear and direct communication and discussion in real-time. The time taken to switch console control from the remote site to the local site and vice versa was under five seconds. The fellow (SB) performed the lymph node dissection independently under remote proctoring. There was a local experienced robotic surgeon (AA) present next to the fellow for safety and he did not have to take-over either. No significant delays were encountered during the switching to cause interference with the mentoring. Average latency during the exercise was 170 and 167 ms, respectively.

RAPN = robotic assisted partial nephrectomy; ms = milliseconds

### Data transmission and network details

Despite the significant round-trip communication distance of 28,000 Km, the overall average latency for the consecutive cases 2 - 5 was 164 ms (minimum 158.9, maximum 196.8) with no delays to impact any of the steps in any of the procedures (Table-2). There were no network failures encountered and the local surgeon (AA) did not have to take-over control from the remote surgeon (SA).

## DISCUSSION

Telesurgery holds significant global healthcare benefits by providing advanced surgical care to underserved and remote areas with limited access (6, 25). Furthermore, it hold immense potential for advancing education and training (15). Over the past two decades, telesurgery progressed from experiments and trials to a real-time surgical option coinciding with the emergence of cost-effective robotic platforms with telecommunication capabilities (26). However, widespread adoption remains limited, especially for cross-border and transcontinental telesurgery, due to challenges relating to network reliability, legal liability, cybersecurity, ethical concerns, cost, and credentialing (27). If expert surgeons, engineers, telecommunication specialists, surgical societies, law makers, company executives, and hospital administrators from around the World get together and establish standardized international guidelines, this could pave the way for safe and effective global implementation of telesurgery (7). The individual publications of human and animal telesurgery experiences contributed significantly to the expansion of telesurgery by allowing societies and hospitals around the World to benefit from it, ultimately leading to the establishment of clear operational frameworks (4, 12). The present study demonstrated the feasibility and reproducibility of different urological telesurgeries performed consecutively with good clinical and technical results. To our knowledge, this is the first cross-border, transcontinental human telesurgery experience in the Middle East, including the first reported telesurgery human RAPN.

Prior to embarking on telesurgery, several elements need to be ensured to guarantee patient safety and successful execution, especially when multiple procedures are planned. A stable network with secure and optimal latency, a fully functional robotic platform with multiple connectivity options, clear and direct audiovisual communication between the local and remote site, compliance with informed consent and legal site-specific protocols, readiness and orientation of the operating room personnel, and clear-cut emergency take-over criteria by the local surgical team (13). In the present study, a telesurgery review committee was responsible for coordinating all the aforementioned in collaboration with the remote team. Their role includes periodic audits of the telesurgery program to ensure safety, reproducibility, and consistency of the outcomes. Telesurgery in the Middle East can help centralize advanced surgical care to reach areas lacking both the expertise and technology and ultimately saving the travel expenses on patients and physicians.

Patel et al. and his group demonstrated the feasibility of telesurgery in animal model across the longest recorded distance between Orlando, Florida, United States of America (USA), and Shanghai, China (approximately 13,000Km) with optimal low-latency connectivity (mean 296 ms) using both 5G and fiber-optic broadband networks (10). They also described telesurgery application in Africa performing two RARPs over two consecutive days using a dedicated fiber-optic connection with a distance of one Km between the surgeon and the patient with a latency < 10 ms (6). In another study, they explored the tele-proctoring potential of telesurgery in a bidirectional manner using porcine models with frequent switching of console control between Shanghai and Orlando with an average latency of 139 ms (15)

The present study between Shanghai and Kuwait sets a compelling example of real-time collaboration between teams from different countries, highlighting how remote surgery can overcome geographical limitations. The successful outcome was the result of extensive connectivity testing achieving an average latency of 166.6 ms and 164 ms for all five cases and for the four consecutive cases 2 to 5, respectively, using a dedicated fiber-optic broadband connection outside peak hours of network congestion. The SD-WAN cloud server played a crucial role in reducing network delay and adding more security and stability (7). Redundancy networks were 5G and dedicated fiber-optic line in Kuwait and Shanghai, respectively (22). During case 1 (December 2024), the average RTL was 181.4 ms with few delays exceeding 300 ms that lasted a second or two. They were not noticeable and did not interfere with any of the steps of the procedure due to instant switching to the redundancy back-up network. During cases 2 – 5 (April 2025), the highest latency obtained was 216 ms and we believe part of the reason for this improvement was using a local server (Shanghai) which helped obtain better latencies (Figure-1). Low RTL minimizes the "move and wait" nature of the procedure. The remote surgeon should be able to synchronize command of an action with visualization of the action on console screen. There was no existing reference value in terms of maximum RTL that is considered optimal for telesurgery, with a range from 100 to 300 ms considered safe to perform the procedure (28). The remote surgeon (SA) adapted well and was not affected by any perceivable delay that could compromise the optimal quality of telesurgery.

Telesurgery can provide the perfect medium for tele-mentoring and tele-proctoring in medical education, emergency situations, areas with restricted access to surgical care, conflict zones, and resource limited setting (3 15). Traditionally, training has been limited by geographical proximity and expert availability. Now experts can mentor and assist in complex procedures remotely. Employing augmented reality tele-mentoring was described previously for COVID-19 positive surgical patients using the ProximieTM platform (29). Moschovas et al. demonstrated robotic tele-mentoring in animal model (15). The present study demonstrated it in human model showing how the remote surgeon (SA) supervised and guided the training fellow (SB) in performing pelvic lymph node dissection during RARP. However, Tele-proctoring requires more trials and clinical validation of the remote site before being accredited as a telesurgery remote training site.

**Figure 4:**
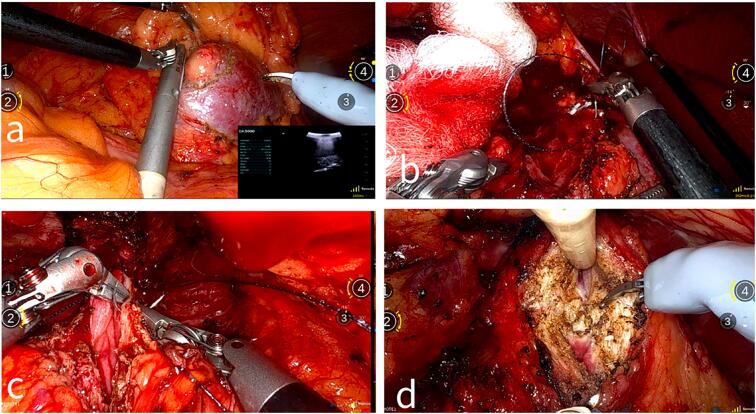
a) Laparoscopic ultrasound drop-in probe used to mark the tumor during right sided RAPN showing real-time ultrasound image at the bottom right corner of the console display. b) Suturing the tumor bed during the final steps of right sided RAPN. c) Urethro-vesical anastomosis during RARP. d) Posterior bladder neck dissection during RARP.

Another valuable demonstration in the present study was showing the feasibility of displaying the real-time intra-operative ultrasound image from a portable ultrasound machine at the patient's location (Kuwait) onto the remote surgeon console display (Figure-4). This was performed during the tumor marking step of the RAPN. This allowed the procedure to feel like local surgery for the remote surgeon, but due to the associated increased risk of bleeding during RAPN, several precautionary steps were taken to ensure patient safety during the telesurgery. A first assistant sparing technique was used where all the sutures and laparoscopic bulldogs were placed inside the peritoneal cavity prior to clamping. After dissection and exposure of the tumor with the aid of intra-operative ultrasound, the renal arteries were clamped using laparoscopic bulldogs to start warm ischemia time. Tumor excision was carried out sharply using the robotic scissors. The tumor bed was over-sewn using 3-0 absorbable barbed suture, 26 mm taper needle with a non-absorbable clip at its tail. Early unclamping and a sliding clip renography were performed using 0 absorbable barbed suture, 37 mm taper needle with a non-absorbable clip at each needle entry and exit on the renal capsule.

RAPN = robotic assisted partial nephrectomy; RARP = robotic assisted radical prostatectomy.

The present study had several limitations. First, the study design with only five patients’ limits generalizing the results on a larger group of patients. Second, the short follow-up prevents assessment of long-term oncological outcomes and comparison with local robotic results. Third, the present study featured selecting non-complex and relatively easy cases which could have introduced selection bias and limited the extrapolation of the findings to patients with more advanced disease. Finally, there was no cost-effective analysis performed which could have been very useful especially when considering the application in resource-limited areas. Despite the aforementioned limitations, the present study achieved an important milestone. The present study demonstrated the concrete achievement of safe and successful execution of multiple human telesurgeries across continents without complications, representing a real-world demonstration of technical feasibility of RARP and RAPN. By continuing to explore and refine this technology we have the opportunity to democratize healthcare. Lessons learned from real-world case studies, including the present study, have provided insights into potential challenges in country to country telesurgery and how to overcome them.

## CONCLUSIONS

The present study demonstrated the feasibility of multiple human telesurgical procedures performed through successful collaboration between two countries from different continents and time zones using a robotic surgical system compatible with fiber optic and 5G connectivity without intraoperative complications and excellent short-term outcomes. Highly reliable, low latency connectivity, and dual network redundancy were essential for the success of the present study. Nevertheless, Telesurgery is still not widely adopted, and one should be aware of the complexity and challenges of applying it to a larger group of patients. There are no specific laws governing international telesurgery, and additional efforts are necessary to further guarantee the safety of large-scale telesurgery in the future. Further improvements in telecommunications are required before larger trials can be carried out with longer follow up. This calls for establishing standardized guidelines and operational framework for cross-border telesurgery.

## ABBREVIATIONS

RARP: = Robotic-assisted radical prostatectomy
RAPN: = Robotic-assisted partial nephrectomy
MTRS: = MicroPort Toumai robotic system
NMPA: = National medical products administration
CE: = Conformite European
RTL: = Round-trip latency
IEPL: = International ethernet private line
SD-WAN: = Software-defined wide-area-network
COVID-19: = Coronavirus disease 2019
BMI: = Body mass index
PIRADS: = Prostate imaging reporting and data system
PSA: = Prostate specific antigen
ISUP GG: = International society of uro-pathology grade group
SHIM: = Sexual health inventory for men
GCC: = Gulf Cooperation Council
IRB: = institutional review board
Km: = kilometer
ms: = milliseconds
mL: = milliliters
GFR: = Glomerular filtration rate
ICU: = Intensive care unit
